# Childhood Maltreatment Is Associated With Aggression Among Male Juvenile Delinquents in China: The Mediating Effects of Callous-Unemotional Traits and Self-Control

**DOI:** 10.3389/fpsyg.2020.01373

**Published:** 2020-06-30

**Authors:** Qinhong Xie, Taiyong Bi, Yan Du, Hui Kou, Bo Yang

**Affiliations:** ^1^School of Criminal Justice, China University of Political Science and Law, Beijing, China; ^2^Center for Mental Health Research in School of Management, Zunyi Medical University, Zunyi, China; ^3^School of Sociology, China University of Political Science and Law, Beijing, China

**Keywords:** juvenile delinquents, aggression, childhood maltreatment, self-control, callous-unemotional traits, mediation effect

## Abstract

**Background:** Aggression is an important risk factor for delinquency and crime in adolescents. Previous studies have indicated that childhood maltreatment plays an important role in the development of aggression. However, whether the effect could be mediated by other factors is still unknown. Evidence suggests that callous-unemotional (CU) traits and self-control may be candidate mediators in the relationship between childhood maltreatment and aggression.

**Methods:** A total of 585 male juvenile delinquents from China were recruited for the present study. We measured self-reported childhood maltreatment, CU traits, self-control, and aggression with the short form of the Childhood Trauma Questionnaire (CTQ-SF), the Inventory of Callous-Unemotional Traits (ICU), the Self-Control Scale (SCS), and the Aggression Questionnaire (AQ), respectively. Furthermore, we constructed multiple mediation models to investigate the mediating effects of CU traits and self-control on the relationship between childhood maltreatment and aggression. Childhood maltreatment and aggression were entered into the model as the independent and dependent variables, respectively, and CU traits and self-control were treated as the mediating variables. In addition, the moderating role of self-control in the relationship between childhood maltreatment and aggression was tested by constructing a moderation model.

**Results:** Correlation analysis showed significant positive correlations among childhood maltreatment, CU traits, and aggression (all *r* values > 0.31, *P* values < 0.01), and self-control was negatively correlated with these three factors (all *r* values < −0.32, *P* values < 0.01). Mediation analyses showed that the relationship between childhood maltreatment and aggression was completely and sequentially mediated by the factors of CU traits and self-control (indirect effect = 0.31, *P* < 0.001). In addition, the relationship between childhood maltreatment and aggression could also be completely mediated by CU traits (indirect effect = 0.24, *P* < 0.001) and self-control (indirect effect = 0.26, *P* < 0.001) separately.

**Conclusion:** Our results indicate that, in a sample of male juvenile delinquents in China, the relationship between childhood maltreatment and adolescent aggression was found to be mediated by CU traits and self-control, which may shed light on the development of aggression among male juvenile delinquents.

## Introduction

Aggression is considered to be a risk factor for subsequent delinquency and crime among adolescents. Evidence shows that the aggression level of juvenile delinquents is higher than that of non-delinquents (Ruchkin et al., 1998; Nas et al., 2005; Barker et al., 2006). Furthermore, high levels of aggression in early life could predict later violent and non-violent delinquency, especially among male adolescents (Roff and Wirt, 1984; Farrington, 1991; Broidy et al., 2003; Tremblay et al., 2004). Therefore, it is important to investigate how aggression develops in adolescents and its risk factors. Childhood maltreatment has been shown to be one of these factors.

Child maltreatment is broadly defined as acts of commission, words or overt actions that cause harm (i.e., emotional, physical, and sexual abuse) and acts of omission, failure to provide for a child’s basic needs or protect a child from harm (i.e., emotional and physical neglect) (Leeb et al., 2008). Numerous studies find a strong relationship between childhood maltreatment and aggression (Chen et al., 2012; Hoeve et al., 2015). Maltreated children are found to show more aggressive behaviors reported by their peers, their teachers, their counselors, and themselves (Shields and Cicchetti, 1998; Bolger and Patterson, 2001; Finzi et al., 2001; Weder et al., 2009; Alink et al., 2012; Shackman and Pollak, 2014; Dackis et al., 2015). Therefore, childhood maltreatment is a well-studied predictor of aggression in children and adolescents. According to a meta-analysis, the incidence rate of childhood maltreatment in China is approximately 54% (Yang et al., 2014). The prevalence of conduct disorder in Chinese male adolescents is 2%∼13% (Liu, 2014). Juvenile delinquents are an appropriate population in which we can examine the relationship between the experience of maltreatment and perpetrated aggression, which is referred to as aggressive behaviors. A better understanding of the association between the experience of childhood maltreatment and aggression is necessary to reveal how conduct disorder develops in Chinese adolescents.

Although the relationship between childhood maltreatment and aggression is well studied, it is still unclear whether other factors could mediate or moderate this effect. Mediation specifies how a given effect occurs. More specifically, the mediating effect explains the mechanism underlying the relationship between the predictive and outcome variables. Assessing mediating effects provides evidence on the predictors first affecting mediators, which, in turn, affect the outcome. A moderator is a variable that affects the relationship between two variables. A significant moderating effect indicates that the impact of the predictor on the outcome varies according to the level of the moderator (Holmbeck, 1997). It is important to investigate mediating and moderating factors. First, investigations on the mediating effect could help to build a more complete theoretical basis to understand the development of aggression. Second, if some factors mediate or moderate the effect of childhood maltreatment on aggression, it is possible to develop intervention methods based on these findings to correct antisocial behaviors among maltreated children. Callous-unemotional (CU) traits and self-control are potential candidate mediating/moderating factors.

According to the fifth edition of the *Diagnostic and Statistical Manual for Mental Disorders* (DSM-5) (American Psychiatric Association, 2013), individuals with high CU traits are often characterized as having a lack of empathy, a lack of guilt, a lack of emotional expression, a lack of caring about one’s performance, and callousness toward others. According to the theory of attachment, individuals learn the schema of close relationships through communication during the early period of childhood (Morton and Browne, 1998). Maltreated children may experience unsafe attachment and have difficulty building a close relationship, which may contribute to low empathy and high CU traits. Consistent with this theory, researchers find that CU traits are significantly correlated with childhood maltreatment, and maltreated participants show higher CU traits than non-maltreated participants (Kimonis et al., 2013b; Dackis et al., 2015). Evidence also shows that CU traits are associated with aggression (Frick and White, 2008; Pasalich et al., 2011; Muñoz and Frick, 2012; Fanti et al., 2013; Kimonis et al., 2013a; Frick et al., 2014), especially among adolescents who were detained or incarcerated (Marsee and Frick, 2007; Kimonis et al., 2014). Furthermore, CU traits are shown to be related to the forms and levels of aggression at a later stage in children with conduct problems, indicating an important role of CU traits in the development of aggression (Frick et al., 2003; Waller et al., 2017). As CU traits are associated with both aggression and childhood maltreatment, CU traits might serve as possible mediators between these two factors. Furthermore, as CU traits are not considered to be a protective factor mitigating the impact of childhood maltreatment on aggression, we did not test the moderational effect of CU traits in the relationship between childhood maltreatment and aggression.

In addition to CU traits, self-control is both a potential mediator and a protective factor between childhood maltreatment and aggression as it has also been shown to be associated with both maltreatment and aggression. Self-control is defined as the effortful capacity of the individual to regulate his or her emotions, thoughts, impulses, or other well-learned or automatic behavioral responses (Hagger et al., 2010). According to the general theory of crime proposed by Gottfredson and Hirschi (1990), parenting factors might impact the development of self-control. Maltreatment is one parenting factor. Therefore, it was proposed that childhood maltreatment may contribute to low self-control (Gottfredson and Hirschi, 1990). Consistently, some evidence shows a relationship between childhood maltreatment and self-control (Pears et al., 2010; Gover et al., 2011; Kort-Butler et al., 2011; Emery et al., 2014). According to Gottfredson and Hirschi (1990) theory, low self-control is the factor that can most directly explain perpetrated behaviors. Consistent with this theory, self-control is shown to be negatively correlated with aggression (Winstok, 2009; Denson et al., 2012; Keatley et al., 2017). Furthermore, individuals with high self-control are less likely to express aggressive intentions or inclinations than those with low self-control (DeWall et al., 2007; Sofia and Cruz, 2015). As self-control is shown to be associated with both childhood maltreatment and aggression, it is possible that self-control plays a mediating role in the relationship between them, which is implied in previous studies. For example, Kort-Butler et al. (2011) find that the association between childhood maltreatment and negative social outcomes, such as criminal behavior, is mediated by low cognitive self-control. Finally, self-control may be a protective factor for the impact of childhood maltreatment on aggression. Studies show that the manipulation of self-control could effectively influence aggressive intention and behavior, implying a causal relationship between self-control and aggression (Finkel et al., 2009). If self-control could moderate the effect of childhood maltreatment on aggression, it implies that the relationship between childhood maltreatment and aggression is dependent on the level of self-control. Interventions against aggression may, thus, be developed through self-control training. It is, thus, important to examine the moderating effect of self-control on the relationship between childhood maltreatment and aggression.

Although we hypothesized that CU traits and self-control may mediate the effect of childhood maltreatment on aggression, it is still unclear how the two factors mediate the effect. From a developmental perspective, interaction and empathy with caregivers contributes to the development of self-regulation during childhood (Kopp, 1982). Therefore, low empathy in individuals with high CU traits may be associated with abnormality in the development of self-control. Empirical evidence also suggests a relationship between CU traits and self-control. For example, individuals with high CU traits are found to report less self-control (Goulter et al., 2017), and individuals with low CU traits are better able to control their behavior (Lotze et al., 2010). Therefore, CU traits and self-control may mediate any effect of childhood maltreatment on aggressive behavior simultaneously. There may be two hypothetic mediation models based on the relationship between CU traits and self-control. If CU traits and self-control could mediate the effect of childhood maltreatment on aggression after controlling the effects for each other, a parallel mediating model is supposed. Otherwise, a sequential mediating model is preferred.

In summary, we examine three issues in the present study. The first motivation of the present study was to examine the relationship between the experience of childhood maltreatment and aggression among a Chinese sample of male juvenile delinquents. The present study only includes male participants because the majority of the individuals in reformatory schools and juvenile correctional facilities in China are boys. To achieve a sufficiently large sample size, it was convenient to conduct the current study on male participants first. The second motivation of the present study was to test the mediating effects of CU traits and self-control on the relationship between childhood maltreatment and aggression. Both the parallel mediation model and the sequential mediation model are tested. In the sequential mediation model, we hypothesized that CU traits are antecedent mediators before self-control. As we mentioned above, theoretical arguments indicate that self-control is directly associated with aggression level (Gottfredson and Hirschi, 1990), and empirical evidence implies a causal relationship between self-control and aggression (Finkel et al., 2009). Therefore, self-control but not CU traits may be appropriately set as the factor close to aggression. In addition, the hypothesis that CU traits and self-control may separately mediate the effect of childhood maltreatment on aggression is also tested through constructing two mediation models with single mediator. Third, we examine whether self-control could moderate the effect of the experience of childhood maltreatment on perpetrated aggression. The results of the present study may shed light on the development of aggression in Chinese adolescents and may help develop intervention methods for antisocial behaviors.

## Materials and Methods

### Participants

The sample comprises 585 male juvenile delinquents from a reformatory school (241) and a juvenile correctional facility (344) in a city in China. Their ages range from 12 to 18 years (mean = 15.88 years, standard deviation (*SD*) = 1.43). Types of offense include affray (9.2%), deliberate injury (9.9%), homicide (1.9%), larceny (29.7%), rape (13.2%), robbery (35.2%), and others (0.9%). A total of 55 (9.4%) and 56 (9.5%) participants reported the deaths of their fathers and mothers, respectively, and 198 (33.8%) and 199 (34.0%) participants reported being away from their fathers and mothers, respectively, for a long period of time because their parents were working in other cities. The fathers of 70 (12.0%) participants and the mothers of 18 (3.1%) participants were reported to have a history of crime or drug addiction.

### Procedures

The present study was approved by the human research ethics committee of Zunyi Medical University and was conducted in accordance with the Declaration of Helsinki. Written informed consent approved by the Human Research Ethics Committee of Zunyi Medical University was given by all the participants and their legal guardians. All participants anonymously completed the Chinese versions of the self-reported questionnaires (20–40 min). They were encouraged to read each item carefully, following the standard guidance from a researcher.

The survey was conducted in quiet classrooms in the two facilities under the supervision of the staff. Each time, no more than 50 participants were asked to fill in the questionnaires simultaneously, and six trained researchers were responsible for checking the completion of answers and addressing the questions raised by the participants. Therefore, no data were excluded due to missing values.

### Measures

#### The Aggression Questionnaire (AQ) (Buss and Perry, 1992)

The original version of the AQ contains 29 items and consists of four subscales: physical aggression (e.g., “I get into fights slightly more than the average person”), verbal aggression (e.g., “I cannot help but getting into arguments when people disagree with me”), anger (e.g., “When frustrated, I let my irritation show”), and hostility (e.g., “I am suspicious of overly friendly strangers”). Each item is rated on a 5-point Likert scale from *1* = *extremely uncharacteristic of me* to *5* = *extremely characteristic of me*. To test the construct validity of the scale, we performed a confirmatory factor analysis (CFA) on the scale. Results show that the four-factor model fit data well (χ^2^/df = 2.67, GFI = 0.93 > 0.90, RMSEA = 0.050 < 0.08), indicating a high construct validity of the scale. The Cronbach’s α coefficient of the total score was 0.89 in undergraduate samples, and the test–retest correlation coefficient was 0.80 (Buss and Perry, 1992). Cronbach’s α was 0.87 in a sample of Chinese adult criminals (Luo et al., 2014). In the present study, Cronbach’s α was 0.88.

#### The Short Form of the Childhood Trauma Questionnaire (CTQ-SF) (Bernstein et al., 2003)

This version of the CTQ contains 28 items (25 clinical items and three validity items) assessing maltreatment history (before 12 years old). The questionnaire consists of five subscales: emotional abuse (e.g., “People in my family said hurtful or insulting things to me”), physical abuse (e.g., “I was punished with a belt, a board, a cord, or some other hard object”), sexual abuse (e.g., “Someone tried to touch me in a sexual way”), emotional neglect (e.g., “There was someone in my family who helped me feel that I was important or special,” reverse coded), and physical neglect (e.g., “There was enough food in the house for everyone,” reverse coded). Response options range from *1* = *never* to *5* = *always*. CFA results show that the five-factor model fit the data well, indicating a high construct validity of the scale (χ^2^/df = 2.21, GFI = 0.93, RMSEA = 0.045). Cronbach’s α was 0.77 in a Chinese sample (Zhao et al., 2005). In the present study, Cronbach’s α is 0.80.

#### The Inventory of Callous-Unemotional Traits (ICU) (Essau et al., 2006)

This scale contains three subscales: callousness, including items about the callous attitude toward others (e.g., “I do not feel remorseful when I do something wrong”); uncaring, including items about the lack of caring about performance (e.g., “I care about how well I do at school or work,” reverse coded); and unemotional, including items about the lack of emotional expression (e.g., “I am expressive and emotional,” reverse coded). Twenty-four items are used to assess the CU traits based on a 4-point scale, from *0* = *not at all true* to *3* = *definitely true*. CFA results show that the three-factor model fit the data well, indicating a high construct validity of the scale (χ^2^/df = 1.61, GFI = 0.93, RMSEA = 0.044). Cronbach’s α was 0.75 in a sample of Chinese adolescents (Yang and Huang, 2013). In the present study, Cronbach’s α is 0.71.

#### The Self-Control Scale (SCS) (Tangney et al., 2004)

This scale contains 36 items and consists of five subscales: self-discipline (e.g., “I never allow myself to lose control”), deliberate/non-impulsive action (e.g., “I’d be better off if I stopped to think before acting,” reverse coded), healthy habits (e.g., “I engage in healthy practices”), work ethic (e.g., “I am able to work effectively toward long-term goals”), and reliability (e.g., “I keep secrets very well”). Items are rated on a 5-point Likert scale ranging from *1* = *not at all like me* to *5* = *very much like me*. The Chinese version of the SCS (Tan and Guo, 2008) consists of five subscales: impulse control, resistance to temptation, healthy habits, concentration on work, and abstinence from entertainment. Generally, the Chinese version of the SCS is not much different from the original scale in the dimensions of impulsive control, healthy habits, and work ethic. However, the dimensions of self-discipline and reliability are replaced with resistance to temptation and abstinence from entertainment due to cultural differences. CFA results show that the five-factor model fit the data well, indicating a high construct validity of the scale (χ^2^/df = 2.70, GFI = 0.94, RMSEA = 0.055). Cronbach’s α was 0.86, and the test–retest reliability was 0.85 in a Chinese undergraduate sample (Tan and Guo, 2008). In the present study, Cronbach’s α is 0.79.

### Statistical Analysis

First, the score of each scale was calculated for each subject. For each subscale, the score of the inverted coded items was recoded. Later, the score of each subscale was calculated by summing all the scores from items belonging to the subscale. Finally, the total score of each scale was calculated by summing all the scores from the subscales. Independent samples *t* tests were used to compare the scores of the scales between groups. Pearson correlation coefficients among the scores of the AQ, CTQ-SF, ICU, and SCS were calculated for both the total scores and subscale scores. Before constructing mediation models, we assessed common method bias through Harman’s single-factor test, which is one of the most widely used techniques to address the issue of common method variance (Podsakoff and Organ, 1986; Podsakoff et al., 2003). First, exploratory factor analysis (EFA) was conducted on the whole questionnaire and each scale. If there is more than one factor in the model and the variance explained by the first factor is relatively low (<40%), a weak impact of the common method on the measures is implied. Next, CFA was conducted on the whole questionnaire and each scale. If the one-factor model does not fit the data well (GFI < 0.9 and RMSEA > 0.08) and the chi-square difference from the original multifactor model is significant (*P* < 0.05), a common method factor may have a limited impact on the measures. In addition, skewness and kurtosis for the scores were calculated to examine the normality of the data. Multicollinearity was then examined through the tolerance and the variance inflation factor (VIF) (Berk, 1977; Chatterjee and Price, 1991).

According to our hypotheses, we constructed five models. In each model, childhood maltreatment was treated as the predictor variable, and aggression was treated as the outcome variable. In the mediation models, CU traits and/or self-control were treated as the mediating variables. In the moderation model, self-control was treated as the moderating variable. Specifically, the five models were as follows: (1) parallel multiple mediation model: CU traits and self-control parallelly mediate the effect of childhood maltreatment on aggression; (2) sequential multiple mediation model: CU traits and self-control sequentially mediate the effect of childhood maltreatment on aggression; (3) mediation model of CU traits: only CU traits mediate the effect of childhood maltreatment on aggression; (4) mediation model of self-control: only self-control mediates the effect of childhood maltreatment on aggression; and (5) moderation model of self-control: only self-control moderates the effect of childhood maltreatment on aggression. The procedure of testing mediation effect is consistent with that proposed by Zhao et al. (2010). The procedure of testing the moderation effect is consistent with that proposed by Coenders et al. (2008). Specifically, childhood maltreatment, CU traits, self-control, and aggression are set as latent variables. Childhood maltreatment consists of five observed variables: physical abuse, sexual abuse, emotional abuse, physical neglect, and emotional neglect. CU traits consist of three observed variables: callous, uncaring, and unemotional. Self-control consists of five observed variables: impulse control, resistance to temptation, healthy habits, concentration on work, and abstinence from entertainment. Aggression consists of four observed variables: physical aggression, verbal aggression, anger, and hostility. Structural equation models (SEMs) were constructed through AMOS^1^. Childhood maltreatment was set as the predictive variable, and aggression was set as the outcome variable. In the mediation models, CU traits and/or self-control were set as the mediators. In the moderation model, self-control was set as the moderator. Mediating effects were tested through a bootstrapping method with a sample size of 5000 and a confidence interval of 95% (Bolin, 2014).

## Results

### Descriptive Statistics and Correlation Analyses

The means and *SD*s of each subscale are displayed in Table 1. Furthermore, we divided participants into two groups based on age (percentile rank over and below 50%). A total of 329 participants were assigned to the younger group (age: 12∼16 years), and 256 participants were assigned to the older group (age: 17∼18 years). The younger group showed higher scores on the scales of childhood maltreatment (physical abuse, sexual abuse, emotional abuse, physical neglect, emotional neglect), CU traits (callousness, uncaring, unemotional) and two of the aggression scales (physical aggression, anger) (all *P* values < 0.05) but lower scores on the three SCSs (resistance to temptation, healthy habits, concentration on work) (all *P* values < 0.05). There was no difference between groups on the following four subscales: verbal aggression, hostility, impulse control, abstinence from entertainment.

**TABLE 1:** Descriptive statistics according to each subscale.

		Score range of each item	Score range of the subscale	*Mean* ± *SD*
Childhood maltreatment	Physical abuse	1–5	5–25	8.74 ± 4.04
	Sexual abuse	1–5	5–25	7.19 ± 2.90
	Emotional abuse	1–5	5–25	9.40 ± 3.75
	Physical neglect	1–5	5–25	9.69 ± 3.80
	Emotional neglect	1–5	5–25	13.42 ± 5.19
Aggression	Physical aggression	1–5	6–30	18.28 ± 6.27
	Verbal aggression	1–5	3–15	7.86 ± 2.42
	Anger	1–5	6–30	14.64 ± 5.43
	Hostility	1–5	6–30	14.69 ± 4.33
Self-control	Impulse control	1–5	6–30	18.92 ± 4.69
	Resistance to temptation	1–5	4–20	11.73 ± 2.72
	Healthy habits	1–5	3–15	10.39 ± 2.66
	Concentration on work	1–5	3–15	9.42 ± 2.09
	Abstinence from entertainment	1–5	3–15	9.11 ± 2.74
CU traits	Callousness	0–3	0–33	11.41 ± 4.92
	Uncaring	0–3	0–24	8.76 ± 3.84
	Unemotional	0–3	0–15	7.44 ± 2.44

*Score ranges of each item and each subscale as well as the mean and standard deviation (*SD*) for each subscale are reported. CU, Callous-unemotional.*

The Pearson correlation coefficients among the 17 subscales of childhood maltreatment, aggression, self-control, and CU traits are illustrated in Table 2. Generally, the subscales of childhood maltreatment, CU traits, and aggression show moderate positive correlations with each other [mean (*r*) = 0.227], and they show moderate negative correlations with the subscales of self-control [mean (*r*) = −0.239].

**TABLE 2:** Correlations among the scores of subscales.

		1	2	3	4	5	6	7	8	9	10	11	12	13	14	15	16	17
Childhood maltreatment (1–5)	1. Emotional abuse	1	0.65**	0.22**	0.45**	0.47**	0.15**	0.14**	0.25**	−0.23**	−0.22**	−0.21**	−0.19**	−0.24**	0.27**	0.12**	0.25**	0.42**
	2. Physical abuse		1	0.24**	0.42**	0.48**	0.14**	0.13**	0.19**	−0.17**	−0.19**	−0.16**	−0.12**	−0.23**	0.22**	0.07	0.19**	0.31**
	3. Sexual abuse			1	0.04	0.14**	0.07	–0.02	0.16**	–0.08	–0.08	–0.06	–0.03	−0.13**	0.05	0.11**	0.12**	0.13**
	4. Emotional neglect				1	0.63**	0.28**	0.21**	0.26**	−0.11**	−0.19**	−0.19**	−0.18**	−0.19**	0.15**	0.01	0.14**	0.22**
	5. Physical neglect					1	0.21**	0.17**	0.28**	−0.12**	−0.22**	−0.17**	−0.20**	−0.18**	0.18**	0.04	0.15**	0.29**
CU traits (6–8)	6. Uncaring						1	0.39**	0.35**	−0.15**	−0.27**	−0.33**	−0.26**	−0.25**	0.29**	–0.03	0.16**	0.07
	7. Unemotional							1	0.19**	–0.05	−0.14**	−0.18**	−0.18**	−0.08*	0.19**	−0.09*	0.04	0.11*
	8. Callousness								1	−0.24**	−0.33**	−0.29**	−0.21**	−0.28**	0.28**	0.06	0.28**	0.25**
Self-control (9–13)	9. Impulse control									1	0.50**	0.27**	0.32**	0.58**	−0.59**	−0.39**	−0.74**	−0.45**
	10. Healthy habits										1	0.27**	0.38**	0.47**	−0.41**	−0.22**	−0.45**	−0.37**
	11. Resistance to temptation											1	0.39**	0.26**	−0.29**	–0.05	−0.25**	−0.21**
	12. Concentration on work												1	0.31**	−0.27**	−0.10*	−0.27**	−0.26**
	13. Abstinence for entertainment													1	−0.53**	−0.27**	−0.48**	−0.38**
Aggression (14–17)	14. Physical aggression														1	0.33**	0.65**	0.39**
	15. Verbal aggression															1	0.41**	0.41**
	16. Anger																1	0.48**
	17. Hostility																	1

*p < 0.05; **p < 0.01. CU, Callous-unemotional.

We further calculate the Pearson correlation coefficients among the four total scores of childhood maltreatment, aggression, self-control, and CU traits. There are positive correlations among factors of childhood maltreatment, aggression, and CU traits (all *r* values > 0.31, *P* values < 0.01). However, the relationships between self-control and other factors were negative (all *r* values < −0.32, *P* values < 0.01).

### Test of Common Method Bias, Normality, and Multicollinearity

Common method bias was tested through Harman’s single-factor method (Table 3). We first conducted EFAs on all the items and each scale. Unrotated EFAs show that KMOs are all higher than 0.8 and that χ^2^ for Bartlett’s test of sphericity are all significant, indicating that the data are suitable for factor analysis. Further analysis shows that, in all the tests, there was more than one factor with an eigenvalue higher than one and that all the first common factors explain no more than 40% of the variance. Next, we conducted CFAs on all the items and each scale. The results show that the one-factor model did not fit the data well (all GFI < 0.9, most RMSEA > 0.08). All the chi-square differences in the original multifactor model are significant. These results may partly address common methodological variance concerns regarding measures used in the present study.

**TABLE 3:** Harman’s single-factor test for common method bias.

Test		Suggested range	All items	ICU	CTQ-SF	AQ	SCS
EFA	KMO	>0.8	0.88	0.80	0.89	0.92	0.86
	χ2		18388.20	1884.04	5027.41	2288.12	4293.86
	*P*	<0.05	<0.001	<0.001	<0.001	<0.001	<0.001
	Number of factors	>1	25	7	6	4	4
	Explained variance by first factor	<40%	14.15%	16.52%	24.71%	32.50%	24.77%
CFA	χ2		1398.55	886.47	1792.40	1143.81	689.21
	df		119	252	275	188	152
	GFI	<0.9	0.73	0.85	0.74	0.79	0.87
	RMSEA	>0.08	0.47	0.07	0.10	0.09	0.08
	Δχ2		962.33	502.62	1238.73	713.01	346.90
	Δdf		17	14	24	17	26
	*P*	<0.05	<0.001	<0.001	<0.001	<0.001	<0.001

*Exploratory factor analysis (EFA) and confirmatory factor analysis (CFA) were conducted on the whole questionnaire and on each scale separately. Note that the suggested range here is the range of value indicating poor fitness of the one-factor model. ICU, Inventory of Callous-Unemotional Traits; CTQ-SF, Short Form of the Childhood Trauma Questionnaire; AQ, Aggression Questionnaire; SCS, Self-Control Scale; KMO, Kaiser-Meyer-Olkin; GFI, Goodness-of-fit index; RMSEA, Root Mean Square Error of Approximation.*

The assumption of a normal distribution was tested by an examination of skewness and kurtosis. It was found that the skewness ranges from 0.13 to 0.68 and kurtosis ranges from 0.005 to 0.50 in the present study (<1), indicating that the data are nearly normally distributed. The assumption of multicollinearity was examined through the tolerance and the VIF. In the present study, the tolerance is 0.77–0.84 (>0.10), and the VIF is 1.20–1.30 (<10), implying sufficiently weak multicollinearities among the factors.

### Model Construction and Testing

First, fit indices were calculated according to the recommendation of Hu and Bentler (1999) and are illustrated in Table 4. As Table 4 illustrates, two models fit the data well: the sequential multiple mediation model and the mediation model of self-control; two models marginally fit the data well (indices fluctuate around the cutoff scores): the parallel multiple mediation model and the mediation model of CU traits; one model poorly fits the data: the moderation model of self-control.

**TABLE 4:** Model fit indices for each model.

Fit index	χ2	df	χ2/df	RMSEA	NFI	IFI	TLI	CFI	GFI
Suggested range				<0.08	>0.9	>0.9	>0.9	>0.9	>0.9
Parallel multiple mediation model	627.08	110	5.70	0.09	0.82	0.85	0.81	0.85	0.88
Sequential multiple mediation model	298.77	99	3.02	0.06	0.91	0.94	0.92	0.94	0.94
Mediation model of CU traits	214.12	42	5.10	0.08	0.84	0.92	0.87	0.91	0.94
Mediation model of self-control	234.50	64	3.66	0.07	0.92	0.94	0.92	0.94	0.95
Moderation model of self-control	8383.32	140	59.88	0.32	0.28	0.28	0.12	0.28	0.65

*Childhood maltreatment and aggression are set as predictive and outcome variables, respectively, in all the models. CU traits and self-control are set as parallel mediators and sequential mediators in the parallel multiple mediation model and sequential multiple mediation model, respectively. The mediation model of CU traits include only CU traits as the mediator. The mediation model of self-control and the moderation model of self-control include only self-control as the mediator and moderator, respectively. RMSEA, Root Mean Square Error of Approximation; NFI, Normed Fit Index; IFI, Incremental Fit Index; TLI, Tucker-Lewis Index; CFI, Comparative Fit Index; GFI, Goodness-of-fit index.*

The parallel multiple mediation model was first tested. The results show that the path from CU traits to aggression is not significant (β = 0.03, *P* = 0.60). Therefore, the parallel multiple mediation model may not be established as the model predicts that both mediators have significant connections with the predictive and outcome variables.

**FIGURE 1:**
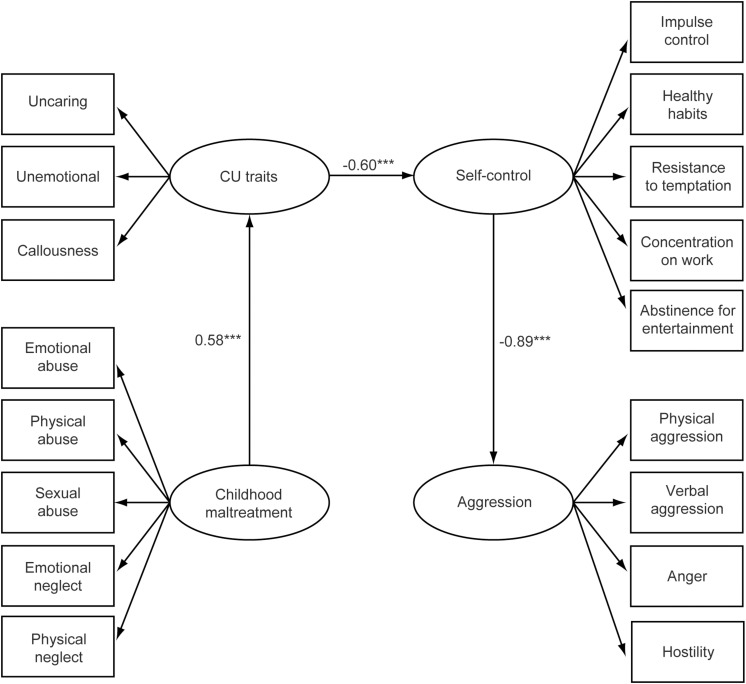
Results from the sequential multiple mediation model. Childhood maltreatment, CU traits, self-control, and aggression are four latent variables in the model. The observed variables that constitute each latent variable are shown in the rectangles. Non-significant paths are omitted, which are the paths from childhood maltreatment to aggression, from CU traits to aggression, and from childhood maltreatment to self-control. ***P < 0.001. CU, Callous-unemotional.

Before testing the sequential multiple mediation model, we tested all pathways from childhood maltreatment to aggression. The results show that three pathways are non-significant: the paths from childhood maltreatment to aggression (β = 0.05, *P* = 0.29), from CU traits to aggression (β = 0.01, *P* = 0.94), and from childhood maltreatment to self-control (β = −0.03, *P* = 0.67). Therefore, we removed these non-significant paths and constructed the sequential multiple mediation model (Figure 1).

Next, we examined the mediating effect in the sequential multiple mediation model (Table 5). First, for the path from childhood maltreatment to self-control, the indirect effect mediated by CU traits is significant (*P* < 0.001), which indicates a significant mediating effect of CU traits in the relationship between childhood maltreatment and self-control. Second, for the path from CU traits to aggression, the indirect mediating effect of self-control is significant (*P* < 0.001), which indicates a significant mediating effect of self-control in the relationship between CU traits and aggression. Finally, for the path from childhood maltreatment to aggression, the indirect effect is significant (*P* < 0.001), which indicates significant mediating effects of CU traits and self-control in the relationship between childhood maltreatment and aggression. Notably, as we mentioned above, the paths from childhood maltreatment to self-control, from CU traits to aggression, and from childhood maltreatment to aggression are all insignificant, indicating that all the mediating effects are complete.

**TABLE 5:** Direct and indirect effects in the sequential multiple mediation model.

Path	Standardized path coefficient	*P*	Confidence interval
**Direct effects**			
Childhood maltreatment → CU traits	0.58	<0.001	[0.50, 0.67]
CU traits → Self-control	−0.60	<0.001	[−0.70, −0.51]
Self-control → Aggression	−0.89	<0.001	[−0.94, −0.84]
**Indirect effects**			
Childhood maltreatment → CU traits → Self-control	−0.35	<0.001	[−0.43, −0.27]
Childhood maltreatment → CU traits → Self-control → Aggression	0.31	<0.001	[0.24, 0.38]
CU traits → Self-control → Aggression	0.54	<0.001	[0.46, 0.62]

*Standardized path coefficients, *p* values, and confidence intervals for the paths in the model are reported. CU, Callous-unemotional.*

The results above reveal a significant pathway from childhood maltreatment to aggression: childhood maltreatment → CU traits → self-control → aggression. Bootstrapping analysis shows that the confidence interval of this path does not include 0, which reveals a significant sequential mediating effect of CU traits and self-control in the relationship between childhood maltreatment and aggression.

Next, we tested the model with a single mediator of CU traits. The direct and indirect effects are illustrated in Table 6. In this model, the direct effect from childhood maltreatment to aggression is non-significant (*P* = 0.399). Other direct effects and the indirect effect are significant (all *P* values < 0.001), indicating that CU traits completely mediate the effect of childhood maltreatment on aggression. Bootstrapping analysis reveals a significant mediating effect of CU traits in the relationship between childhood maltreatment and aggression.

**TABLE 6:** Direct and indirect effects in the mediation models with a single mediator.

Model	Path	Standardized path coefficient	*P*	Confidence interval
Mediation model of CU traits	**Direct effects**			
	Childhood maltreatment → Aggression	0.06	0.399	[−0.14, 0.24]
	Childhood maltreatment → CU traits	0.54	<0.001	[0.40, 0.66]
	CU traits → Aggression →	0.44	<0.001	[0.25, 0.65]
	**Indirect effects**			
	Childhood maltreatment→ CU traits → Aggression	0.24	<0.001	[0.13, 0.41]
Mediation model of self-control	**Direct effects**			
	Childhood maltreatment → Aggression	0.05	0.162	[−0.02, −0.13]
	Childhood maltreatment → Self-control	−0.28	<0.001	[−0.38, −0.16]
	Self-control → Aggression	−0.93	<0.001	[−0.98, −0.88]
	**Indirect effects**			
	Childhood maltreatment→ Self-control → Aggression	0.26	<0.001	[0.15, 0.36]

*Standardized path coefficients, *p* values, and confidence intervals for the paths in the model are reported. CU, Callous-unemotional.*

Similarly, we constructed a mediation model with a single mediator of self-control. The direct and indirect effects are also illustrated in Table 6. In this model, the direct effect from childhood maltreatment to aggression is non-significant (*P* = 0.162). Other direct effects and the indirect effect are significant (all *P* values < 0.001), indicating that self-control completely mediates the effect of childhood maltreatment on aggression. Bootstrapping analysis reveals a significant mediating effect of self-control in the relationship between childhood maltreatment and aggression.

Finally, the moderating effect of self-control was tested. Results show that the interaction of childhood maltreatment and self-control could positively predict aggression (β = 0.13, *P* < 0.001, CI = [0.01, 0.24]), indicating a significant moderation effect of self-control on the relationship between childhood maltreatment and aggression.

## Discussion

In the present study, we explore the relationship between childhood maltreatment and aggression among male juvenile delinquents in China and the mediating and the moderating effects of CU traits and self-control in this relationship. Comparing with other samples, the present results show that CU traits are generally higher in our sample (callousness: 11.41; uncaring: 8.76; unemotional: 7.44) than in a non-delinquent sample (callousness: 5.13; uncaring: 6.08; unemotional: 4.31) (Luo, 2015); aggression is much higher in our sample (physical aggression: 18.28; verbal aggression: 7.86; anger: 14.64; hostility: 14.69) than in a non-delinquent sample (physical aggression: 2.25; verbal aggression: 1.63; anger: 2.31; hostility: 2.19) (Ge and Zhao, 2010). These results reflect the high CU traits and aggression levels in our participants. Next, the results show that childhood maltreatment is positively associated with aggression. Importantly, this relationship is completely mediated by CU traits and self-control. Interestingly, only the sequential mediating effect of childhood maltreatment → CU traits → self-control → aggression is significant, and other paths from childhood maltreatment to aggression are non-significant. Furthermore, the results from mediation models with a single mediator showed that both CU traits and self-control could completely mediate the effect of childhood maltreatment on aggression. These results reveal a possible developmental process from childhood maltreatment to aggression (i.e., childhood maltreatment may first predispose an individual to developing CU traits, which, in turn, decrease self-control, and, in turn, enhance aggression). These findings may provide evidence of the mechanisms underlying the development of aggression, especially in the population of male juvenile delinquents.

Consistent with previous studies showing a close relationship between childhood maltreatment and aggression (Connor et al., 2004; Chen et al., 2012; Kolla et al., 2013; Hoeve et al., 2015), we also find a significant positive correlation between the two factors. More importantly, the results not only support the relationship between them but also demonstrate the specific way in which childhood maltreatment affects aggression. According to our results, the effect of childhood maltreatment on aggression is completely mediated by CU traits and self-control. This finding implies that the relationship between childhood maltreatment and aggression may be indirect. If we can manipulate an individual’s state of CU traits and self-control, childhood maltreatment may not necessarily impact one’s aggression. However, CU traits may be difficult to change. Evidence shows that CU traits are relatively stable from mid- to late adolescence (Forsman et al., 2008). Furthermore, intervention in conduct-disordered adolescents is found to be ineffective for individuals with high CU traits (Waschbusch et al., 2007). Comparatively, self-control is easier to change, and there are effective ways to manipulate self-control. For example, Finkel et al. (2009) depletes the self-regulatory resources of participants via an attentional control task and bolsters their self-regulatory strength via self-regulatory regimens. Such manipulations significantly affect participants’ aggression responses. In addition, a recent study demonstrates a larger effect of self-control training on males than on females (Friese et al., 2017). Therefore, the aggression of Chinese male juvenile delinquents might be reduced by self-control training even if they have high levels of CU traits. The present results reveal the important role of self-control on the relationship between childhood maltreatment and aggression, which may provide a crucial piece of evidence on the effect of self-control training in forensic rehabilitation programs. Future studies may investigate whether the manipulation of self-control could affect the relationship between childhood maltreatment and aggression.

In addition to the development of aggression, our results also shed light on the development of CU traits in male juvenile delinquents. The results show that childhood maltreatment is positively associated with CU traits, which is consistent with the findings of previous studies (Kimonis et al., 2013b; Dackis et al., 2015; Walters, 2018). These findings indicate possible ways to prevent the development of high CU traits. On the one hand, if the experience of childhood maltreatment increases the level of CU traits, a possible method to prevent high CU trait development could be intervening in the maltreatment. For example, parental warmth, concern, and love might be important protective factors against CU traits. Some results indeed show that CU traits could be lowered by family based interventions, such as parent and child therapy and social learning–based parent training (Hawes et al., 2014). These interventions were found to enhance parental warmth and, thus, improve the CU traits of the children (O’Connor et al., 2013). On the other hand, if children with high CU traits are raised in an abusive environment, interventions should involve both parents and children.

Another interesting finding of the present study is the negative correlation between CU traits and self-control. Previous studies mostly explore the relationship between CU traits and aggression as well as the relationship between self-control and aggression. However, little is known about the relationship between CU traits and self-control. Some evidence shows that individuals with high CU traits report less self-control (Goulter et al., 2017), and individuals with low CU traits are better able to control their behavior (Lotze et al., 2010). In contrast, another study did not find a significant relationship between a lack of guilt and impulsivity, which are core components of CU traits and self-control, respectively (Mann et al., 2018). In the present study, we find that CU traits are significantly correlated with self-control, indicating a close relationship between these factors. Individuals with high CU traits are relatively insensitive to punishment cues and, thus, may show less risk avoidance than controls (Blair et al., 2001). Additionally, individuals with high CU traits may be insensitive to others’ fearful and sad emotions (Dadds et al., 2006; Kimonis et al., 2006; Dawel et al., 2012). The lack of risk avoidance and empathy may further lead to maladaptive behaviors characterized by risk seeking and uncontrolled behaviors, which might be the reason why CU traits show a negative correlation with self-control. More studies are needed to further elucidate the relationship between CU traits and self-control.

Finally, our results show that self-control may moderate the effect of childhood maltreatment on aggression. However, it should be noted that the moderation model of self-control poorly fit the data, making it hard to conclude whether self-control could moderate the relationship between childhood maltreatment and aggression. As a result, we may only confirm that self-control could completely mediate the relationship between childhood maltreatment and aggression. These results demonstrate that the effect of childhood maltreatment on aggression might occur through the impact of self-control, implying that self-control manipulation might be an effective intervention for aggression. However, it is still unclear whether the impact of childhood maltreatment on aggression depends on the level of self-control. It is necessary to further test the moderating role of self-control on the relationship between childhood maltreatment and aggression in another sample.

## Limitations

First, the present study mainly focuses on male juvenile delinquents. Thus, the findings may not easily generalize to other populations, such as females and adolescents without antisocial behaviors. Evidence shows that the relationship between childhood maltreatment and aggression may be different for girls and boys (Cullerton-Sen et al., 2008). Future studies are needed to examine the present results in other populations, especially in female adolescents. Second, in addition to the four factors examined in the present study, other factors that may affect the results should also be considered in further studies. For example, in the present study, aggression is divided into the four dimensions of physical aggression, verbal aggression, anger, and hostility. However, aggression could also be divided into proactive and reactive aggression. Evidence shows that proactive aggression is more related to delinquency, psychopathic personality, and serious violent offending, and reactive aggression is more associated with impulsivity and hostility (Raine et al., 2006). There may be different results for proactive aggression and reactive aggression. In addition, other criminogenic or psychosocial factors may also affect the present results. For example, the mediating effect may depend on education level, peer relationships, and parenting styles. Finally, it should be noted that the data-collection setting might have affected participants’ responses. For example, in the present study, a group of participants completed the questionnaire simultaneously in a single room and were asked to complete all the items. Although informed consent was obtained, some of them may respond carelessly to some items to complete the questionnaire as soon as possible. Future studies may improve the data collection setting and avoid the influence of response bias.

## Conclusion

The present study demonstrates that childhood maltreatment, CU traits, self-control, and aggression are significantly correlated with each other in Chinese male juvenile delinquents. A multiple mediation model further shows that childhood maltreatment is positively associated with aggression, which is completely mediated by CU traits and self-control. These results shed light on the development of aggression and CU traits.

## Data Availability Statement

The raw data supporting the conclusions of this article will be made available by the authors, without undue reservation, to any qualified researcher.

## Ethics Statement

The studies involving human participants were reviewed and approved by the Human Research Ethics Committee of Zunyi Medical University. Written informed consent to participate in this study was provided by the participants’ legal guardian/next of kin.

## Author Contributions

QX conceived of the study, participated in its design, questionnaire distribution and data analysis, and drafted the manuscript. TB and YD participated in coordination, questionnaire distribution, and statistical analysis. HK and BY participated in study design and revised the manuscript critically for important intellectual content. All authors contributed to the article and approved the submitted version.

## Conflict of Interest

The authors declare that the research was conducted in the absence of any commercial or financial relationships that could be construed as a potential conflict of interest.

## Footnotes

1 www.statisticssolutions.com/amos
